# Modeling and Experimental Validation of the VARTM Process for Thin-Walled Preforms

**DOI:** 10.3390/polym11122003

**Published:** 2019-12-03

**Authors:** Da Wu, Ragnar Larsson, Mohammad S. Rouhi

**Affiliations:** 1 Division of Material and Computational Mechanics, Department of Industrial and Materials Science, Chalmers University of Technology, SE-412 96 Göteborg, Sweden; dawu@chalmers.se; 2 Division of Materials and Production, RISE SICOMP, SE-431 22 Mölndal, Sweden; mohammad.rouhi@ri.se

**Keywords:** liquid composite molding, porous media theory, process modeling, fiber preform deformation, resin flow

## Abstract

In this paper, recent shell model is advanced towards the calibration and validation of the Vacuum-assisted Resin Transfer Molding (VARTM) process in a novel way. The model solves the nonlinear and strongly coupled resin flow and preform deformation when the 3-D flow and stress problem is simplified to a corresponding 2-D problem. In this way, the computational efficiency is enhanced dramatically, which allows for simulations of the VARTM process of large scale thin-walled structures. The main novelty is that the assumptions of the neglected through-thickness flow and the restricted preform deformation along the normal of preform surface suffice well for the thin-walled VARTM process. The model shows excellent agreement with the VARTM process experiment. With good accuracy and high computational efficiency, the shell model provides an insight into the simulation-based optimization of the VARTM process. It can be applied to either determine locations of the gate and vents or optimize process parameters to reduce the deformation.

## 1. Introduction

The need for fiber-reinforced polymer composite (FRPC) structures is immense among various industries, as the pursuit of lightweight design and sustainable development becomes the social focus. The applications are not only in the traditional fields of transportation, infrastructure, and sports, but also medicine [1] and battery technologies [2]. Different manufacturing processes have emerged to fabricate high-performance FRPC laminates effectively and affordably, e.g., Injection Molding, Sheet Forming, and Tape Lay-Up processes for thermoplastic FRPCs or Autoclave and Liquid Composite Molding (LCM) processes for thermoset composite materials. In many production scenarios, the FRPC parts can be considered as thin-walled structures, i.e., the in-plane dimensions are significantly larger than the thickness. The Vacuum-assisted Resin Transfer Molding (VARTM) process is usually selected to produce this type of FRPC parts, due to the benefits of low volatile organic compounds emission, high flexibility and scalability, and good quality [3].

The VARTM process integrates fiber placement, resin injection, and cure in one step [4]. In the course of the VARTM process, the dry fiber preforms made from fabrics are stacked on the single-sided tool, a plastic vacuum bag seals and covers preforms. The resin is then infused into preforms by the vacuum inside the bag. The main challenges of the VARTM processes are air entrapment and preform deformation. Air entrapment [5] can lead to macro- or micro-voids; the lubrication effect [6] and the relaxation mechanism [7] affect the thickness and fiber volume fraction distribution of final parts. For the last couple of decades, to overcome potential defects, researchers have started developing models to assist the design of the mold and process parameters.

Many methods have been carried out to monitor and characterize the resin flow, e.g., SMART-weave system [8], linear direct current [9], fiber optics [10], point voltage sensors [11], and dielectric sensors [12]. As a member of the Liquid Composite Molding process family, the VARTM process can be modeled as the fluid flow in porous media and formulated in the continuum mechanics framework. Hammami and Gebart [13], Correia et al. [14], and Fracassi and Donadon [15] reported 1-D analytical models of the saturated flow. Various unsaturated flow models are also reported, e.g., [16,17,18]. Some researchers formulated multiphase flow equations of porous media with the absence of capillary pressure [19,20]; others include the capillary effect, e.g., [21,22,23,24,25,26]. Another approach is to consider the flow as a dual-scale flow. The intra-tow flow can be treated as delayed infiltration of tows, which can be modeled as sinks of inter-tow flow, as described in [27,28,29,30,31,32,33]. In addition, a multiscale algorithm has been reported by Tan and Pillai [34].

Researchers used various methods to characterize the preform deformation during VARTM processes, e.g., the linear variable differential transducer [35,36], structured light scanner [37], and digital image correlation (DIC) [38]. To model the dynamically coupled flow moving and preform deforming, Acheson et al. [39] modeled the preform as a nonlinear spring bed. Li et al. [40] implemented an auxiliary preform compaction model to predict the thickness when the infusion is just finished. Bayldon and Daniel [41] reported a saturation depended preform thickness model. Raounak et al. [42] developed a multi-layer model. Rubino and Carlone [43] suggested an integrated structural-fluid dynamic approach.

Nowadays, industries aim to provide superior quality and reasonably priced products to establish a niche amid the keen market competition. The process modeling has been applied to optimize the process plan, e.g., the determination of inlet and outlet positions [44], or used to adjust process parameters for active control in real-time alongside the streamline [45]. Although the presented models can successfully replicate experiments, either some of the models cannot handle the complex geometry, or other models need further information of the through-thickness permeability that is difficult to measure; neither model can be easily applied to the process optimization. Because of the nature of the thin-walled preform, it is assumed that the through-thickness flow can be ignored, and the preform deforms only along the normal of mold, as reported in [46]. The shell model finally simplified the VARTM process to a problem of 2-D in-plane resin flowing in the 3-D deformable preform. In this regard, the proposed model can present the primary physical phenomena of the VARTM process with much smaller numbers of degrees of freedom than full 3-D models.

In Section 2, we present the mathematical model of the VARTM for thin-walled parts. The experiment is introduced in Section 3, followed by numerical simulation in Section 4. Furthermore, the results from the experiment and model are compared and discussed in Section 5. Finally, the concluding remarks are made in Section 6.

## 2. Model of Resin Infusion in the Deformable Preform

In this section, we outline the model of resin infusion in a deformable preform based on the theory of porous media. The model consists of three parts: (1) the concept of the saturation degree; (2) the kinematics of the (un-)saturated thin-walled preform; and (3) the constitutive relations of the fiber preform compaction, the capillary pressure, and the in-plane Darcy velocity.

### 2.1. Saturation Degree

**Figure 1:**
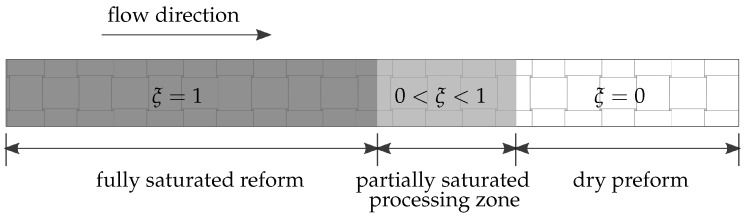
A sketch of fully saturated, partially saturated, and dry regions of the preform during the vacuum-assisted resin transfer molding (VARTM) process.

During the resin infiltration, three regions are observed [27]: the dry preform, a processing zone where preform is partially saturated, and a full-saturated part that expands from inlet to the process zone as shown in Figure 1. The concept of saturation degree is introduced to locate those regions. Let $n^{s}$ and $n^{f}$ represent the volume fraction of fibers and fluid (a mixture of liquid and air), respectively, which are constrained by

(1) $$n^{s}+n^{f}=1\;.$$

The fluid phase consists of two constituents: the liquid resin and the air, which are further distinguished by the saturation degree, 0≤ξ≤1, as

(2) $$n^{f}=\varphi^{l}+\varphi^{g}=\xi n^{f}+\left(1-\xi \right)n^{f}\;,$$

where $\varphi^{l}\xi n^{f}$ denotes the liquid volume fraction and $\varphi^{g}\left(1-\xi \right)n^{f}$ is the air volume fraction. From the homogenization [25], the fluid density is expressed as

(3) $$\rho^{f}=\xi \rho^{l}+\left(1-\xi \right)\rho^{g}\;,$$

where $\rho^{l}$ and $\rho^{g}$ are the intrinsic densities of the liquid and air, respectively. With the same homogenization approach, the fluid pressure *p* is obtained as

(4) $$p=\xi p^{l}+\left(1-\xi \right)p^{g}\;,$$

where $p^{l}$ and $p^{g}$ are the pressures of the liquid and the air, respectively.

### 2.2. Kinematics of the (Un-)Saturated Thin-Walled Preform

**Figure 2:**
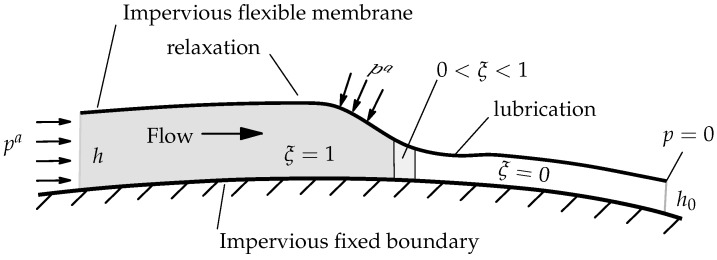
The VARTM process on a deformable thin-walled preform.

Figure 2 shows a principal sketch of the VARTM process. The preform top surface undergoes the atmospheric pressure $p^{a}$ beneath a flexible membrane; the bottom is fixed to the mold.

**Figure 3:**
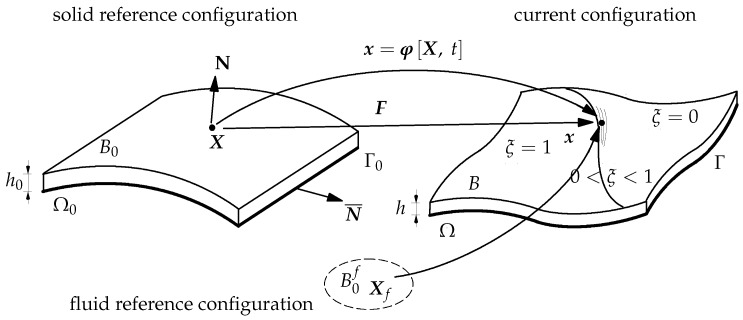
Configurations of the partially saturated preform. $B_{0}$ and $B_{0}^{f}$ are the reference configuration of solid and fluid, respectively. The bottom of $B_{0}$ is denoted as $\Omega_{0}$ that is bounded by the boundary $\Gamma_{0}$. N and $\bar{N}$ are the out-of-plane normal and the in-plane normal, respectively. The material particle X in $B_{0}$ and $X_{f}$ in $B_{0}^{f}$ occupies the same position x in the current configuration *B*. The bottom of the current configuration is named as Ω surrounded by Γ.

To describe the kinematics of the thin-walled preform, we consider the configurations of the solid (preform) and fluid (liquid and air) in Figure 3. The thin-walled preform is considered as a single director shell surface $\Omega_{0}$ with the normal N. Furthermore, we assume that the preform can compress or expand only along the director N, as discussed in [46]. To measure the deformation, we define the stretch λ as the ratio between the thicknesses of the deformed preform, *h*, and the undeformed preform, $h_{0}$, i.e.,

(5) $$\lambda :=\frac{h}{h_{0}}\;.$$

Thus, the deformation gradient tensor F can be expressed as

(6) F=I+(λ−1)N⊗N,

where I is the 2nd-order identity tensor. Evidently, the volume change of the preform is J=detF=λ.

### 2.3. In-Plane Darcy Flow

Let $v^{f}$, $v^{l}$, and $v^{g}$ denote the fluid velocity, liquid velocity, and gas velocity, respectively. The solid velocity v is defined by $\dot{\varphi }$, where $\dot{•}$ represents the material time derivative correlating with $B_{0}$. We introduce the relative velocities of the fluid, liquid, and gas as

(7) $$v^{rf}=v^{f}-v,\;v^{rl}=v^{l}-v,\;\mathrm{and}\;v^{rg}=v^{g}-v.\;$$

Therefore, the Darcy velocities $v^{\mathrm{df}}$, $v^{\mathrm{dl}}$, and $v^{\mathrm{dg}}$ are defined as

(8) $$v^{df}=n^{f}v^{rf},\;v^{dl}=\varphi^{l}v^{rl},\;\mathrm{and}\;v^{dg}=\varphi^{g}v^{rg}.$$

Finally, from the work in [25], the fluid Darcy flux is formulated as

(9) $$\rho^{f}v^{\mathrm{df}}=\rho^{l}v^{\mathrm{dl}}+\rho^{g}v^{\mathrm{dg}}\;.$$

The masses of solid, fluid, and liquid substances can be formulated as: $M^{s}=Jn^{s}\rho^{s}$, $M^{f}=Jn^{f}\rho^{f}$ and $M^{l}=J\varphi^{l}\rho^{l}$, respectively. The principle of mass conversation for the solid phase yields

(10) $${\dot{M}}^{s}=\;0\;,\;$$

for the fluid phase

(11) $${\dot{M}}^{f}+J\left(\rho^{f}v^{\mathrm{df}}\right)\cdot \nabla =\;0\;,$$

and for the liquid constituent

(12) $${\dot{M}}^{l}+J\left(\rho^{l}v^{\mathrm{dl}}\right)\cdot \nabla =\;0\;.$$

Summarizing Equations (10) and (11), we obtain the pressure equation as

(13) $$\dot{J}\rho^{f}+J\left(\rho^{l}-\rho^{g}\right)n^{f}\dot{\xi }+J\left(1-\xi \right)n^{f}{\dot{\rho }}^{g}+J\left(\rho^{f}v^{\mathrm{df}}\right)\cdot \nabla =0\;.$$

From Equation (12), we obtain the saturation equation

(14) $$Jn^{f}\dot{\xi }+\dot{J}\xi +Jv^{\mathrm{dl}}\cdot \nabla =0\;.$$

### 2.4. Constitutive Relations

#### 2.4.1. Fiber Preform Compaction

Toll [47] reported a fiber preform compaction model, which is an exponential law related to the fiber volume fraction $n^{s}$. According to this packing law, the free energy of a hyperelastic preform can be defined as

(15) $$\psi \left[J\right]=\frac{k^{s}E{\left(n_{0}^{s}\right)}^{m}}{m-1}\left(J^{1-m}-m+\left(m-1\right)J\right)\;,\;$$

where $k^{s}E$ and *m* are parameters in Toll’s packing law. From (15), the effective pressure $p^{e}$ is obtained as

(16) $$p^{e}=-\frac{\partial \psi }{\partial J}=k^{s}E{\left(\frac{n_{0}^{s}}{J}\right)}^{m}\left(1-J^{m}\right)\;.$$

On the other hand, from the Terzaghi’s equation and [48], the total stress of the impregnated preform $\hat{\sigma }$ correlates with the effective preform stress σ and the fluid pressure *p* as

(17) $$\hat{\sigma }=\sigma -pB\;,$$

where B is the Biot tensor, which is a diagonal second-order tensor,

(18) $$B=b_{1}E_{1}⊗E_{1}+b_{2}E_{2}⊗E_{2}+b_{3}N⊗N\;,$$

in the inertial Cartesian coordinate, where $b_{1}$, $b_{2}$, and $b_{3}$ are determined by fiber assembly. For example, $b_{1}=b_{2}=b_{3}=b\approx 1$ for most isotropic fiber preform, see [49].

**Figure 4:**
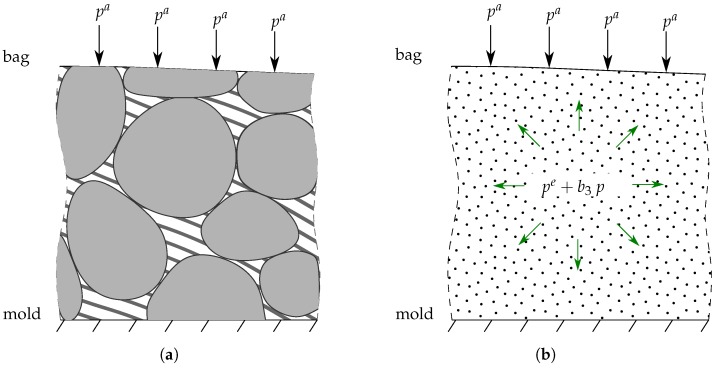
The real (un-)saturated preform composed of solid and fluid phases is averaged statistically to a smeared body. The applied atmospheric pressure is balanced out by the combination of effective pressure and fluid pressure. (**a**) A sketch of the real impregnated fiber preform; (**b**) A sketch of the smeared impregnated fiber preform.

During the VARTM process, the atmospheric pressure, the effective pressure, and the fluid pressure hold equilibrium, as shown in Figure 4. The effective pressure can be derived from the principle of virtual work as

(19) $$p^{e}\left[\lambda \right]=k^{s}E{\left(n_{0}^{s}\right)}^{m}\left(\lambda^{-m}-1\right)=p^{a}-b_{3}\;p\;.$$

From Equation (19), we obtain an explicit constitutive relation of the stretch,

(20) $$\lambda ={\left(1+\frac{p^{a}-p\;b_{3}}{k^{s}E{\left(n_{0}^{s}\right)}^{m}}\right)}^{-1/m}\;.$$

#### 2.4.2. Capillary Pressure and Universal Gas Law

Brooks and Corey [50] reported a phenomenological capillary model,

(21) $$p^{c}\left[\xi \right]=p^{g}-p^{l}=p^{ent}\;\xi^{-\frac{1}{n_{b}}}\;,$$

where $p^{ent}$ denotes the entry pressure and $n_{b}>0$ controls the shape of the capillary pressure curve. Including Equation (21) in Equation (4), we find the expressions of the intrinsic liquid and gas pressures in terms of the fluid pressure and the saturation degree as

(22) $$\begin{array}{cc} p^{l} & =p-\left(1-\xi \right)p^{c}\;,\\ p^{g} & =p+\xi p^{c}\;. \end{array}$$

Using the universal gas law, we obtain the compressible gas density $\rho^{g}$

(23) $$\rho^{g}=k^{g}p^{g}=k^{g}\left(p+\xi p^{c}\right)\;\mathrm{with}\;k^{g}=\frac{m^{g}}{RT}\;,$$

where *R* is the universal gas constant and *T* is the absolute temperature.

#### 2.4.3. In-Plane Darcy Resin Flow in the Unsaturated Preform

In this study, the main focus is the VARTM process of thin-walled fiber preforms. Given the nature of thin-walled preforms, the through-thickness flow is omitted, as discussed in [46]. By making this assumption, we can reduce the number of degrees of freedom of the problem. For example, if the full 3-D flow is considered, at least three nodes are needed: one at the bottom, one on the top, and one in between. However, in a shell model, there is only one node needed through the thickness. As the through-thickness flow is neglected, the 3-D flow turns to a 2-D flow. From the theory of porous media, we know the Darcy flow velocity as

(24) $$\begin{array}{cc} v^{dl} & =-k_{rl}\frac{\hat{k}}{\mu_{l}}\nabla p^{l}\;,\;\\ v^{dg} & =-k_{rg}\frac{\hat{k}}{\mu_{g}}\nabla p^{g}\;,\; \end{array}$$

where $\mu_{l}$ and $\mu_{g}$ are the liquid and gas viscosity, respectively. The relative permeabilities $k_{rl}$ and $k_{rg}$ are defined by Burdine [51]

(25) $$\begin{array}{cc} k_{\mathrm{rl}} & =\xi^{3+\frac{2}{n_{b}}}\;,\;\\ k_{\mathrm{rg}} & ={\left(1-\xi \right)}^{2}\left(1-\xi^{1+\frac{2}{n_{b}}}\right)\;. \end{array}$$

$\hat{k}$ is the intrinsic permeability tensor of the preform. For a 2-D “thin-walled” porous media problem, $\hat{k}$ is a second-order tensor, which is composed of four permeability $k_{ij}$ in each direction of the planar preform, where i=1,2, and j=1,2. The permeability highly relate to the volume fraction of solid $n^{s}$ or porosity of the preform $n^{f}$. Let ${•}_{0}$ represents values of the undeformed preform, and • represents values of the deformed preform, from the Kozeny–Carman equation, we have

(26) $$k_{ij}=J^{-2}{\left(\frac{1-n_{0}^{s}}{J-n_{0}^{s}}\right)}^{-3}k_{ij,0}\;.$$

## 3. Experiments

### 3.1. Materials

Non-crimp fabrics (NCFs) that fabricated from Toho Tenax^®^ HTS45 E23 carbon fiber are used in the experiment as the preform. To remove the influence of curing, we choose the rapeseed oil to replace the thermoset resin. The properties of the fabric and resin materials used in the experiment are listed in Table 1.

### 3.2. Experiment Set-Up and Process

**Figure 5:**
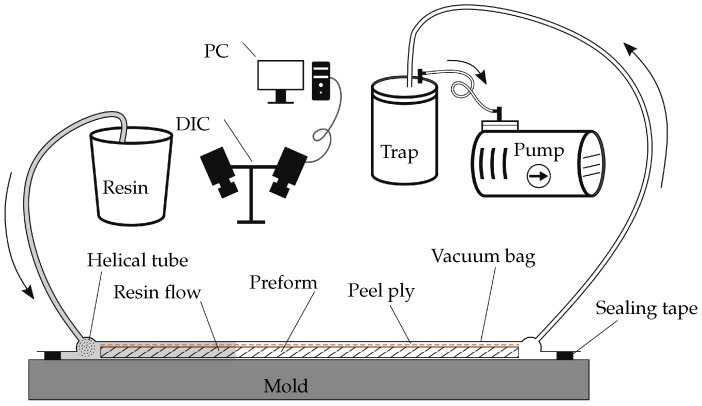
The VARTM experiment setup.

The experimental setup is depicted in Figure 5. First, eight layers of NCF are stacked on a flat aluminum mold (1 m × 1 m), following the [−45/45/90/0]${}_{s}$ layup, to assemble the fibrous preform (22 cm × 38 cm). Then, on top of the preform, a layer of peel ply is placed. Because the preform is thin (6.5 mm) compared with other dimensions, the distribution layer is ignored. A helical tube is placed at the inlet of the mold. The inlet tube connects to the resin reservoir, and the outlet tube is connected to the vacuum pump. Finally, the mold is covered by a vacuum bag and sealed with tacky tapes around all edges of the preform. When the VARTM experiment setup is ready, the Digital Image Correlation (DIC) system from GOM Aramis^®^ is installed on top of the mold to carry out the in situ monitoring during the infusion process.

When the experiment starts, the inlet valve is clamped, and the outlet valve is open to pump out the air. The vacuum stays at 32 mbar for 15 min until infusion starts. Once the vacuum is ready, the resin flows into the mold from the reservoir under the atmospheric pressure. Meanwhile, the DIC is monitoring the resin flow development and the deformation of the preform. The data acquisition lasted 800 s after the experiment started.

## 4. Numerical Simulation

**Figure 6:**
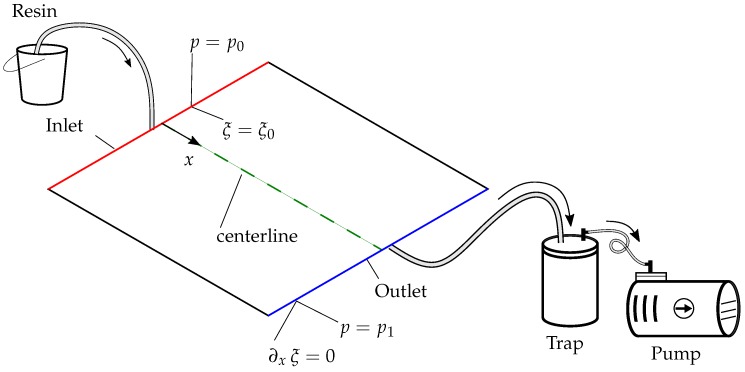
The setup of the numerical simulation of the VARTM process.

The setup of the numerical simulation of the experiment is shown in Figure 6. The red line indicated the resin inlet, and the blue line is the outlet. The black edges are impermeable. The green centerline is selected to collect and represent results from the simulation.

**Figure 7:**
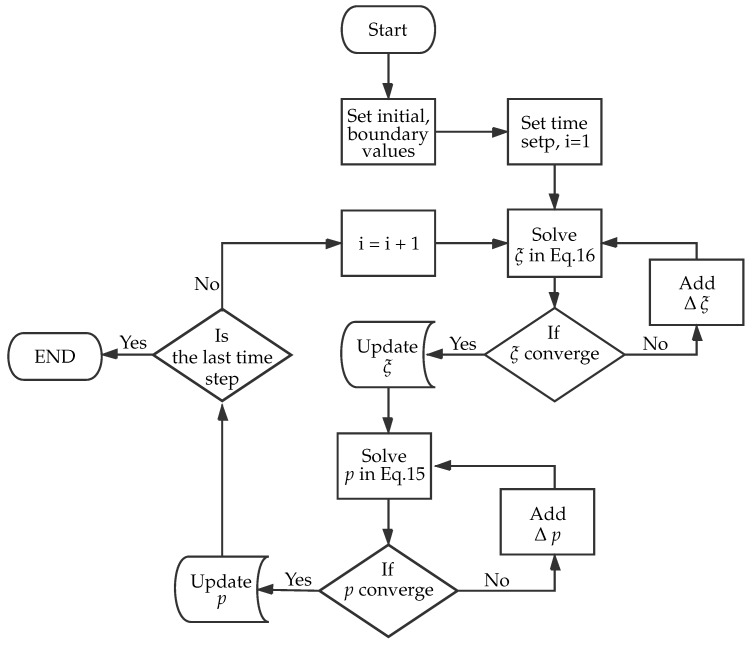
A flow chart illustrates the staggered numerical approach.

During the VARTM process, the fiber volume fraction changes due to the preform deformation. The preform deformation interferes with the permeability of the preform, it affects the resin flow and interacts with the fiber volume fraction again. Thus, the fluid pressure, *p*, and the saturation degree, ξ, are strongly coupled, which makes the problem complicated. The Equations (13) and (14) are solved by using the finite element method as a boundary value and initial value problem developed in [25,46]. Figure 7 shows the staggered approach to decouple the problem.

At the inlet, the pressure is $p_{0}$ = 1 atm and the saturation degree is given as $\xi_{0}$ = 1.0. At the outlet, pressure and saturation degree are determined by $p_{1}$ = 32 mbar and $\partial_{x}\xi$ = 0, respectively; the initial values are set to ${}^{0}\xi =\xi (x,0)$ = 0.001 and ${}^{0}p=p(x,0)$ = 32 mbar. The initial value ${}^{0}\xi$ has been chosen small enough, but it avoids numerical singularity.

All of the parameters listed in Table 1 and Table 2 will be used in the simulation. The air viscosity $\mu_{g}$, the atmospheric pressure $p^{a}$, the molecular mass of the air $m^{g}$, and the universal gas constant *R* are intrinsic parameters. The initial preform thickness $h_{0}$, initial fiber volume fraction $n_{0}^{s}$, resin density $\rho^{l}$ and viscosity $\mu_{l}$, permeability tensor ${\hat{k}}_{0}$, and absolute temperature *T* are measured before starting mold-filling.

The capillary pressure exponent $n_{b}$, the entry pressure $p^{ent}$, the packing stiffness of the fiber preform $k^{s}E$, and the packing law exponent *m* are calibrated from the result of the experiment at 29 s. To implement the calibration, we define the objective function in terms of the preform deformation as

(27) $$f=\frac{1}{2}\sum_{i=1}^{n}{\left(\Delta h_{sim,i}-\Delta h_{exp,i}\right)}^{2}\;,$$

where $\Delta h=h-h_{0}$ is the change of thickness. The subscripts “sim,i” and “exp,i” represent the simulation and experiment results at the ith point (*n* points in total) along the centerline, respectively. When calibrating the parameters, we discretized the prefrom to 500 four-node bilinear elements and chose the time step size as 0.005 s.

**Figure 8:**
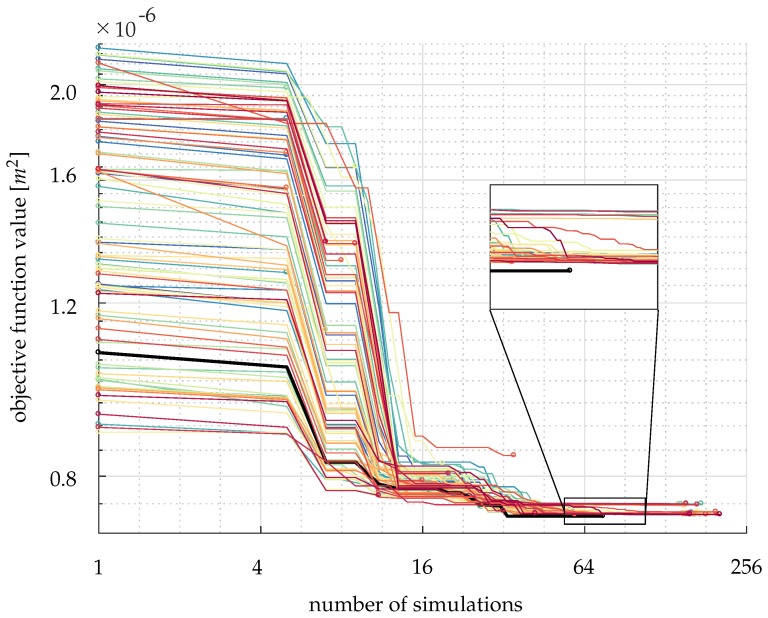
The history of the reduction of objective function values for each guess of parameters. The black line identifies the group of initial guess that gives the lowest objective function value. The circles represent the start and end of the course of each the minimum objective function search.

One-hundred start guesses of the parameters are randomly generated from the Latin hypercube sampling. The Nelder–Mead Simplex algorithm [52] is used to find the minimum value of the objective function, with the initial simplex size of 5%. To identify which guess ends up with the minimum objective function value, we plot the evolution of objective function values of each guess in Figure 8. The black line gives the lowest objective function value, and the parameters calibrated from that specific guess are chosen and listed in Table 2.

To validate the model, we simulated the experiment from the start of infusion to 800 s based on the measured and calibrated parameters. The mesh size in the simulation is the same as that used in the calibration, but the time step size changes to 0.001 s to improve the stability of the solution.

## 5. Results and Discussion

**Figure 9:**
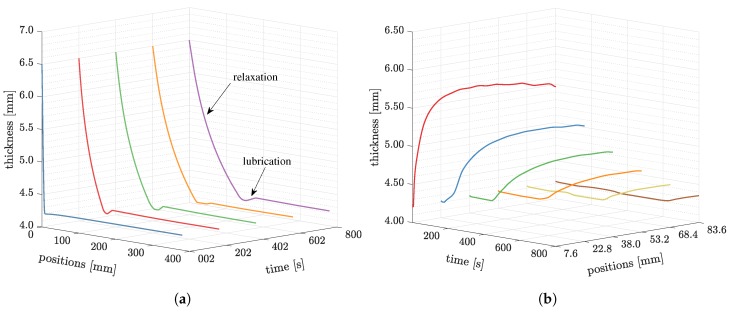
The thickness changes at different times and positions from the simulation. (**a**) The thickness variations along longitudinal direction at 2, 202, 402, and 800 s; (**b**) The thickness development at position *x* = 7.6, 22.8, 38.0, 53.2, 68.4, and 83.6 mm.

We plot the profiles of the preform at the centerline (in Figure 6) to illustrate the preform deformation during the process in Figure 9a. At 2 s, the whole preform compacts to 4.2 mm thick, except at the constrained inlet. As the resin gradually infiltrates the preform, the fluid pressure balance against the atmospheric pressure. Consequently, the pressure applied to the preform will be relieved, and the relaxation [7] takes place. As shown in Figure 9a, the relaxation is followed by a local pit, which is called the lubrication effect [6]. At the flow front, the liquid resin lubricates fiber preform and reduces the friction between fibers. This leads to the reduction of the preform stiffness. We can observe a small region where the preform is further compressed. From 2 s to 800 s, increasingly more resin flows into the preform, and consequently, the flow front moves forwards further. The lubrication region moves together with the flow front, and a larger relaxation region is developed meanwhile.

**Figure 10:**
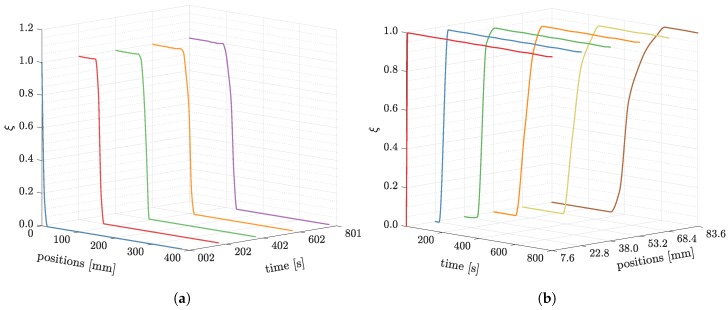
The saturation degree changes at different times and positions from the simulation. (**a**) The saturation degree profiles along longitudinal direction at 2, 202, 402, and 800 s; (**b**) The saturation degree developments. Positions *x* = 7.6, 22.8, 38.0, 53.2, 68.4, and 83.6 mm away from the inlet are selected.

In Figure 10b, we plot the preform thickness up to 800 s at different locations. Along the centerline, six positions were picked, which are 7.8, 22.8, 38.0, 53.2, 68.4, and 83.6 mm away from the inlet, respectively. The preform at 7.8 mm expands to a stable thickness very fast in an exponential way. The positions where are far away from the inlet keep compacted until the resin front arrives. Once the flow arrives, the preform will start to expand. Moreover, the rate of change of preform expansion decreases as the distance from the inlet increases, i.e., the curve is flatter.

In Figure 10a, the saturation degrees from the simulation are plotted. At the beginning of the process (e.g., at 2 s), the curves of saturation degree are very steep and almost appear discontinuous. The discontinuity of saturation degrees implies that the width of the process zone (see Figure 1) is small. However, in the course of the process, the process zone turns wider later. In other words, the curve of saturation degree will represent a smoother transfer from zero to one. Figure 10b depicts the development of saturation degrees at specific points in the whole 800 s infusion. It is obvious when the saturation curves turn from abrupt to smooth.

**Figure 11:**
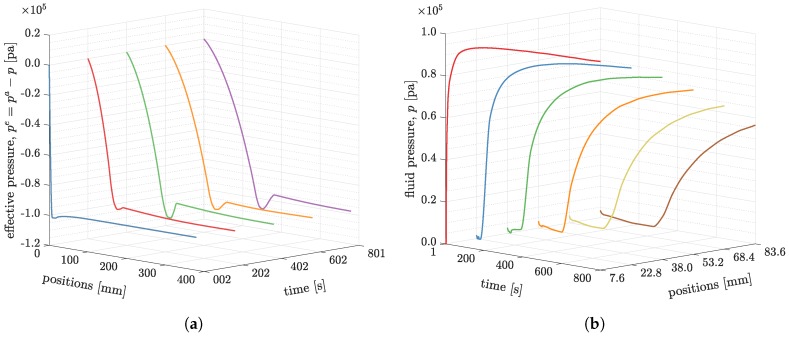
The pressure changes at different times and positions. (**a**) The pressure profiles along longitudinal direction at 2, 202, 402, and 800 s; (**b**) The pressure developments at position *x* = 7.6, 22.8, 38.0, 53.2, 68.4, and 83.6 mm.

The effective pressure profiles in Figure 11 are similar to the ones shown in Figure 9. The pressure curves drop to the bottom; and then jump to a local peak; and, finally, gradually decrease to the applied vacuum, as shown in Figure 11a. The fluid pressure profiles are plotted in Figure 11b. It shows that the rate of change of fluid pressure is high at where it is close to the inlet, and the rate is low at where it is far from the inlet. At positions x=22.8 mm and x=38.0 mm, there are some pressure oscillations when the process just started, as the blue and green curves show in Figure 11b.

**Figure 12:**
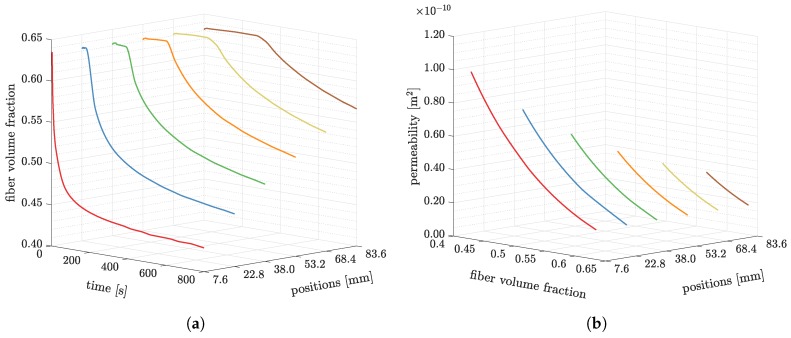
The pressure changes for different times and positions. (**a**) The fiber volume fraction changes at position *x* = 7.6, 22.8, 38.0, 53.2, 68.4, and 83.6 mm; (**b**) The permeability *k*_11_ changes at position *x* = 7.6, 22.8, 38.0, 53.2, 68.4, and 83.6 mm.

As a consequence of the preform deformation, the fiber volume fraction will change, and the permeability will also vary as the architecture of the preform has been updated. We elucidate the change of the fiber volume fraction and the permeability variation in Figure 12. From Figure 12a, we can observe that the fiber volume fraction reduces in the course of the process. The fiber volume fraction at where is close to the inlet reduces faster (red curve) than where it is far from the inlet (brown curve). Consequently, a smaller time step size is adopted.

To represent how the permeability changes with the volume fraction, we plot the permeability along the longitudinal direction, $k_{11}$, in Figure 12b. As the fiber volume fraction increases, the permeability decreases. On the other hand, the rate of change of pressure also decreases in the course of the process. Consequently, the process becomes slower and slower, until eventually the infusion is finished.

**Figure 13:**
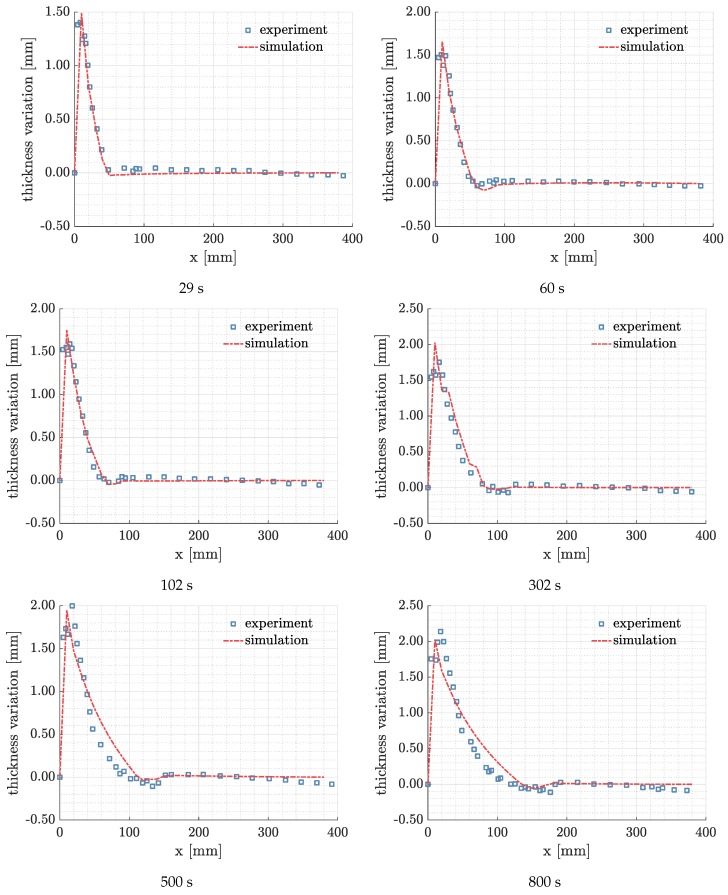
The comparison of thickness variation between the experiment and simulation.

To validate the model, we compare the results of simulation with the experiment through a series of times. Because the thickness variation, $\Delta h=h-h_{0}$, fluctuates at the early stage of the process, we selected denser times at the beginning and looser times in the end. In Figure 13, the simulation results show good agreements with the experimental results, especially for the locations of the maximum relaxation and lubrication. From 29 to 302 s, the relaxation from the simulation almost keeps in line with the experiment. However, at 500 s and 800 s, there are some deviations between the simulation and the experiment. The neglected bending of the preform may cause the deviations, especially when the relaxation zone is wide. Nevertheless, the simulation can still give a good match of the lubrication location and the corresponding compression. To identify the difference between the simulation and the experiment, we design the error, $e_{s}$, as

(28) $$e_{s}=\left|\sum_{i=1}^{n}\frac{\Delta h_{sim,i}-\Delta h_{exp,i}}{\Delta h_{exp,i}}\right|\times 100\%\;,$$

where *n* is the total number of measured thickness variation. $\Delta h_{sim,i}$ and $\Delta h_{exp,i}$ have been defined in Equation (27). Table 3 lists the $e_{s}$ at different times. The largest error is below 15%, and the minimum error is ~2%. Thus, the model yields a generally good estimation of the process regarding the thickness variation.

**Figure 14:**
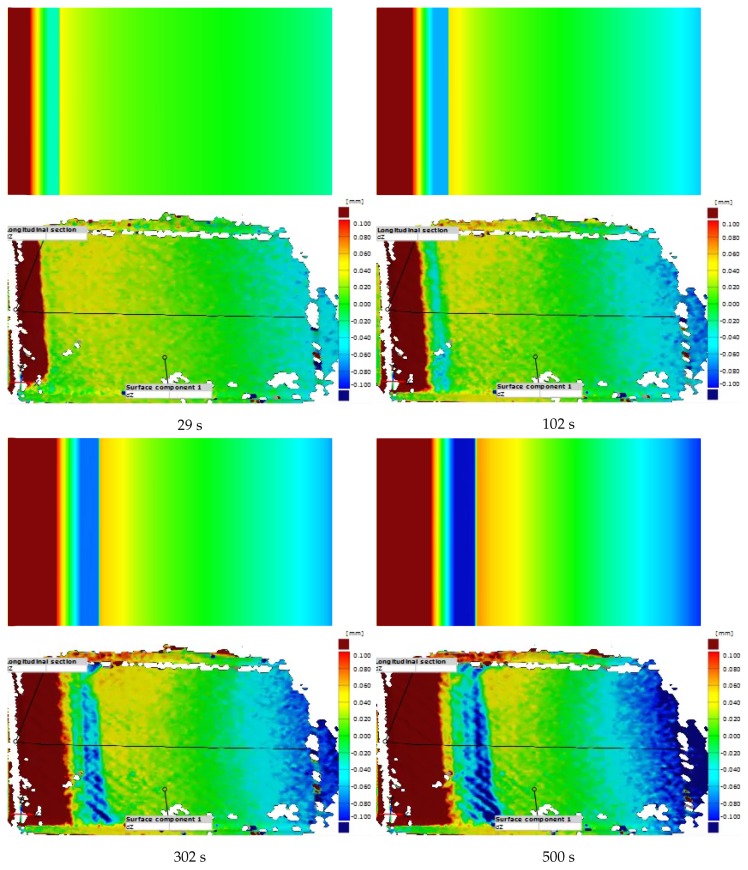
The comparison of thickness variation between simulation contours and Digital Image Correlation (DIC) images. The warm colors represent the expansion, and the cold colors denote the compression.

Finally, the DIC images and the simulation contours of Δh are compared in Figure 14. The simulation contours are placed on the top, and the DIC images are put at the bottom. Both of DIC images and contour plots are divided into different bands of color. For instance, the contour plot at 102 s has been divided into a red band, followed by a narrow yellow band and a green band. A light sapphire band appears after the green band, and a yellow-to-green transition band is followed. Another light sapphire band locates on the right side of the plot.

In conclusion, the DIC images and simulation contours resemble each other in both the band locations and widths. Further, on both the DIC images and the simulation contours, the transitions of color between bands can be observed similarly. From this comparison, the model shows the capability to simulate the preform thickness variation in the course of the VARTM process.

## 6. Conclusions

In this study, the shell model in [46] has been validated with a VARTM process experiment on the thin-walled preform. The model considers the resin flow as a problem of multiphase flow in porous media. The through-thickness flow is omitted, and the preform is treated as a shell. Thus, the flow is assumed to the in-plane flow. Furthermore, the preform deformation is simplified to either the expansion or the compression along the normal of its surface. To these regards, the process changes from a 3-D problem to a 2-D problem. The number of degrees of freedom of the problem is dramatically reduced. For example, the eight nodes tetrahedral element used in 3-D can be replaced by four nodes shell element, which will reduce half of the number of degrees of freedom. To validate the model, we carried out a mold-filling experiment. A flat plate is infused by rapeseed oil using the VARTM process. A DIC system records the deformation of the preform. On the other hand, a simulation of the experimental process is performed. Through the comparison between the experiment and simulation, the results show that the model can predict the preform thickness evolution with limited errors, especially at the beginning of the filling. Nevertheless, it shows larger errors at the late stage of the process. It can be explained by the neglected bending effect in the present model. However, the simulation gives values and positions of the maximum relaxation and compression similar to the experimental results. Moreover, the model successfully mimics the preform relaxation mechanism and lubrication effects. In sum, the present model, with the assumptions of in-plane flow and shell kinematics, provides good predictions of the preform deformation in the VARTM process and needs less computational efforts.

## Figures and Tables

**Table 1:** Fabric and resin properties.

Item	Identification	Value
fabric	longitudinal permeability, ${\hat{k}}_{11}$	$1.15\times 10^{-10}$ m${}^{2}$
	transverse permeability, ${\hat{k}}_{22}$	$1.26\times 10^{-10}$ m${}^{2}$
	fiber volume fraction, $n_{0}^{s}$	0.411
resin	density, $\rho^{l}$	930 kg/m${}^{3}$
	viscosity, $\mu_{l}$	0.067 Pa · s

**Table 2:** Parameters of the preform, resin, and environment.

Parameters	Unit	Identification	Value
$h_{0}$	[m]	preform thickness	6.5 $\times 10^{-3}$
$\mu_{g}$	[Pa· s]	gas viscosity	1.983 $\times 10^{-5}$
$n_{b}$	-	capillary pressure constant	4.5
$p^{ent}$	[Pa]	entry pressure	$1.02\times 10^{5}$
$p^{a}$	[Pa]	atmospheric pressure	$1.01325\times 10^{5}$
$m^{g}$	[kg/mol]	gas molar mass	$2.897\times 10^{-2}$
*R*	[J/K· mol]	ideal gas constant	8.314
*T*	[K]	absolute temperature	293
$k^{s}E$	[Pa]	packing stiffness	$2.66\times 10^{6}$
*m*	-	packing law exponent	6.0

**Table 3:** $e_{s}$ at each selected time.

Time [s]	29	60	102	302	500	800
$e_{s}$ [%]	8.81	2.91	2.01	13.42	8.91	6.89
